# Investigating the Effect of the Environment on Prey Detection Ability in Humans

**DOI:** 10.1038/s41598-019-43797-0

**Published:** 2019-05-15

**Authors:** Peter J. Allen, Jan M. Wiener, Christos Gatzidis, Chris B. Stringer, John R. Stewart

**Affiliations:** 1 Department of Creative Technology, Faculty of Science and Technology, Bournemouth University, Talbot Campus, Fern Barrow, Poole, BH12 5BB UK; 2 Department of Psychology, Ageing and Dementia Research Centre, Bournemouth University, Talbot Campus, Fern Barrow, BH12 5BB Poole, UK; 3 CHER, Department of Earth Sciences, Natural History Museum, SW7 5BD London, UK; 4 Department of Life and Environmental Science, Faculty of Science and Technology, Bournemouth University, Talbot Campus, Fern Barrow, BH12 5BB Poole, UK

**Keywords:** Behavioural ecology, Biological anthropology

## Abstract

Visual search experiments used in the field of psychology may be applied to investigate the relationship between environments and prey detection rates that could influence hunting behaviours in ancient humans. Two lab-based experiments were designed to examine the effects of differing virtual environments, representing Marine Isotope Stage 3 (MIS3) in Europe, on participants’ ability to locate prey. The results show that prey detection performance is highly influenced by vegetation structure, both in terms of the biome type (wooded vs. grassland environments) and the density of the vegetation (trees in wooded and shrubs in grassland environments). However, the density of vegetation has a greater relative effect in grassland than in wooded biomes. Closer examination of the transition between biomes (relative percentages of trees vs. shrubs) at the same vegetative density shows a non-linear relationship between prey detection performance and the relative tree to shrub percentages. Changes in the distribution of biomes occurred throughout the Quaternary. The composition of those biomes will have likely affected hominin hunting behaviours because of their intermediary effects on prey detection performance. This may, therefore, have played a role in the turn-overs of hunter-gatherer hominin populations during MIS3 and at other times in the Quaternary.

## Introduction

Once hominins (whether archaic *Homo* or anatomically modern humans) had spread from Africa and colonised Eurasia, they would have been more exposed to the climatic and environmental oscillations known to have taken place on Milankovitch and sub-Milankovitch timescales^1,2^. Climate change on these scales has been documented in faunal and palynological data, where it was seen affecting vegetation dynamics in Europe^3–6^. Throughout the last 500,000–600,000 years, Eurasia has been occupied by a series of distinct hominin cultures and/or biological populations^7,8^. The nature of the transitions or replacements between these is unclear, although competing hypotheses have been presented. These hypotheses can generally be categorised either as “continuous”, which is to say that a culture developed into another over time^9^, or “discontinuous”, where a culture is replaced by an entirely separate population of people^10^.

During the Milankovitch and sub-Milankovitch climate oscillations, vegetation varied from more open during relatively cold episodes, to more closed, during warmer times^5^. Dansgaard-Oeschger events^1^ (D-O events) in particular feature periods of rapid warming, followed by more gradual cooling and have been linked to changes in the composition of the paleoecology of Europe^4,5^, and it is possible that hominin populations possessed relevant behavioural adaptations to living in these distinct environmental conditions. The kind of behavioural change that would have been necessary because of such environmental changes is likely to have included adopting differing hunting behaviours. Hunting techniques are thought to vary according to vegetative density, with the two extremes being woodland, which when relatively dense is hypothesised to involve encounter hunting, while in more open grassland/tundra habitats a more pursuit-orientated mode of hunting is practised^11–14^.

It would seem likely that prey visibility is a factor that would influence the hunting methods used in different vegetational environments. This may be an as-yet unexplored facet of hominin cultur/biological transitions. We therefore designed two experiments that utilised 3D computer graphics technology to construct virtual environments modelled to resemble southern France during Marine Isotope Stage 3 (MIS3), a stage known for cultural and biological transitions in addition to rapid climatic shifts^15–17^. The aim of both experiments was to investigate the effects of environmental change on the prey detection component of hunting behaviour, which in turn may have implications for the hominin transitions at this time. The first experiment investigated the effects of increasing vegetative density on the ability of participants to detect a prey animal (a red deer *Cervus elaphus*) within both wooded and grassland biomes. The second experiment investigated the effects of an environment transitioning between grassland and wooded on prey detection ability. The tree and shrub models used in our virtual environments were obtained via Interactive Data Visualizations’ Speed Tree software, the colouring and texture is achieved using high quality photographs of their real-world counterparts and the models are realistically sized. We do not see any reason to assume the same species of trees and shrubs would have looked any different during MIS3, and therefore assume that our virtual environments are a realistic proxy for the MIS3 environments they represent.

In standard visual search experiments, participants attempt to identify whether a target item is present amongst a number of distractor items (for example to locate the target letter T amongst the distractor letter L’s). Adding distractors results in a linear increase in participant response time in these types of experiments^18^. It is reasonable to assume that the vegetation in our experiments will have a distractor effect as participants attempt to locate the deer. In traditional visual search experiments the distractors do not occlude the target but rather serve as ‘false targets’. In our more naturalistic settings however there is the potential for vegetation to either partially or completely hide the target object, as would occur in the real world.

A gradual change in prey detection ability with changing vegetative density and composition could support the idea that a culture may have adapted gradually over time to their changing environment. If, on the other hand, the change is abrupt then hominins may not have been able to respond fast enough and hence moved or died out. This could help shed some light on the nature of hominin transitions during MIS3 in particular, and more generally across Milankovitch and sub-Milankovitch climate change.

In line with the requirements for pursuit-hunting^12^, we hypothesised that participants would be able to detect prey at much further distances in an open grassland environment, as compared to a closed wooded biome, where both biomes are populated with the same number of vegetation objects. We also hypothesised that there would be a drop in average detection distance as the vegetative density in both types of environment increased, due to the distractor effect of the vegetation objects in the environments and the tendency of these objects to obscure the prey (either partially or completely).

## Methods

### Experiment 1

#### Materials

**Figure 1:**
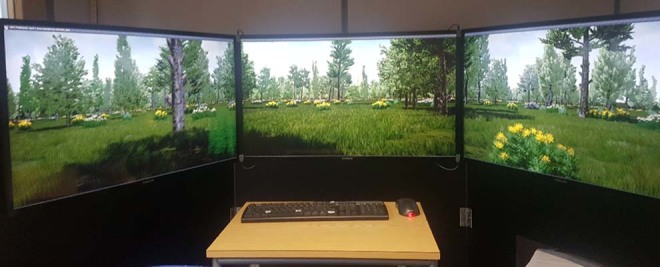
The 3-screen setup in the Bournemouth University Psychology Department. The two side screens are angled inwards at 60 degrees, giving a 180-degree horizontal field of view across all 3 screens.

Participants interacted with a desktop application which presented 3D virtual environments via a 3-screen setup in the Bournemouth University Psychology Department (Fig. 1). The application was created using Epic Games’ Unreal Engine 4.10, a popular contemporary third-party developer game engine.

**Figure 2:**
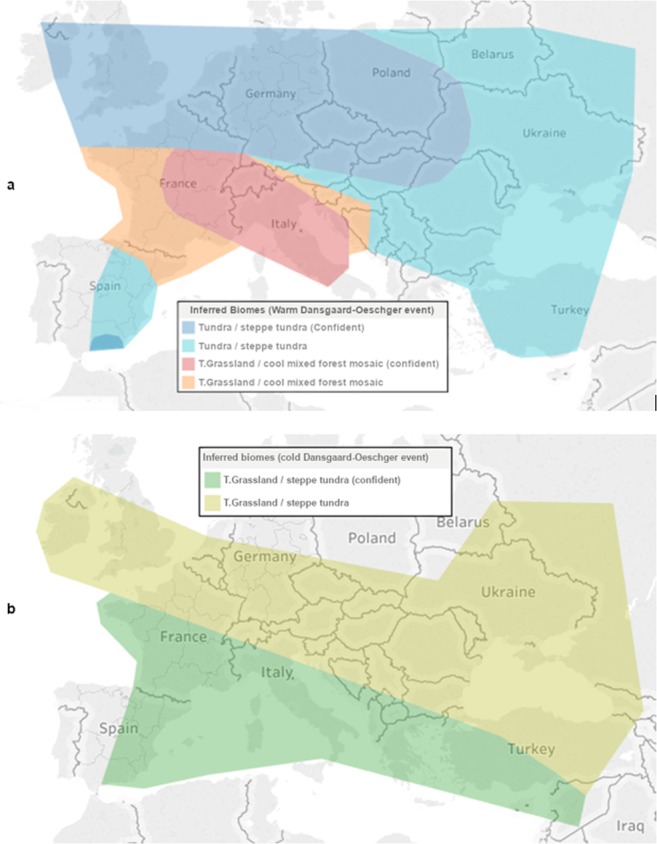
Inferred distribution of biomes in MIS3 Europe. (**a**) Inferred biome distribution in Europe during a warm Dansgaard-Oeschger event^5^, and (**b**) during a cold Dansgaard-Oeschger event^5^ “T.” = Temperate.

**Figure 3:**
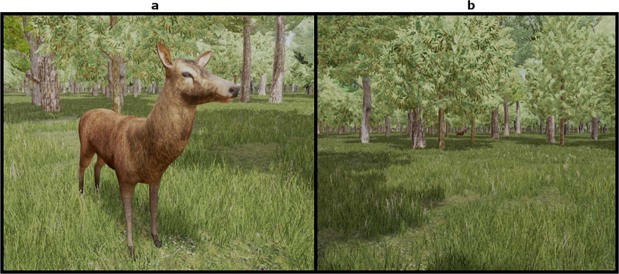
The red deer model utilised in the experiment. As viewed up close (**a**) and the same shot with the camera moved further back to show how the deer begins to become lost amongst the trees despite, in this case, being fully visible (**b**).

The virtual environments contained several floral objects which were either trees (Juniper, Spruce, and Scots Pine) or shrubs (*Valeriana*, Wormwood and St. John’s Wort). These specific taxa were chosen based on the palynological data obtained from MIS3 southern France concerning periods of relative warmth and cool^4^ (Fig. 2). Each environment also contained a target object (3D model of a red deer). The specific 3D model^19^ for the deer was chosen for its accuracy in likeness to a real red deer both in form and coloration (Fig. 3), having been created by a taxidermist for the purpose of being anatomically accurate. Initially, the deer model featured antlers which were removed to avoid a potential visual pop-out effect^20,21^. When placed in our environments the deer model was uniformly scaled to be 180 cm in length and 110 cm to the shoulder in height, placing it within realistic physical dimensions of both male and female red deer^22^. The shrub models had height ranges from 105 cm to 145 cm depending on the taxa, which meant none were capable of completely occluding the deer model. The trees had trunk diameters in the range of 30 cm to over 1 m, depending on the species and size of tree. This meant most trees could completely occlude the deer if its mesial plane was oriented perpendicular to the participant’s view (is viewed head-on), but could not if the deer was oriented laterally with respect to the participant’s view (is viewed side-on). The effect of different light levels was not part of this experiment and so all scenes used the same lighting setup. This setup was similar to the default bright daytime lighting that the Unreal Engine provides but since static lighting was not used we inserted non-shadow casting directional “fill lights” to avoid having unrealistically dark areas of shade.

**Figure 4:**
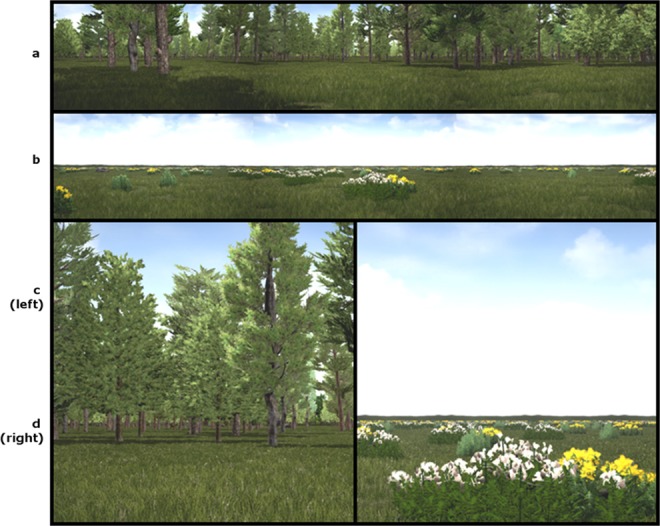
Typical environments used in Experiment 1. (**a**) Typical wooded virtual environment. (**b**) Typical grassland virtual environment. (**c**,**d**) Close up views of the environments shown in (**a**,**b**).

The biomes used in this experiment were wooded environments and grasslands. Either biome could have vegetation objects at a density of 2000, 5000, 8000 or 11000 objects per square kilometre (trees in the wooded areas and shrubs in the grasslands, Fig. 4). Each environment also contained 50 rocks (each ~180 cm tall) at a density of 200 per km^2^ to help break up the environment and ensure that the wooded environments contained other objects in addition to the trees and the deer. In the real world, such rocks may exist due to severe flood events or glaciers^23^. Without these rocks the participants would automatically deduce that any object in the scene that was not a tree must be the deer without them having to pay any attention to it. In the grassland environment the distant shrubs could also easily be mistaken for the deer.

Each environment was square in shape with an area of 0.25 km^2^. The environment was then populated with the required tree or shrub models and, finally, the deer model. The participant started in the centre of one of the edges of the environment facing inwards across it.

During a pilot experiment, we arrived at the average distances at which participants were able to locate the deer in each environment type and density combination, and also the maximum correct spotting distance at which any participant responded throughout the whole pilot. In the real experiment, the deer was placed at approximately 50 m further than the overall maximum correct spotting distance from the pilot in half of the trials (max distance trials), or, in the other half, at approximately 50 m further than the maximum correct spotting distance from the pilot for the given environment and density combination (non-max distance trials). This was done in order to determine whether placing the deer closer to the participant start location in order to reduce trial duration influenced the results.

**Figure 5:**
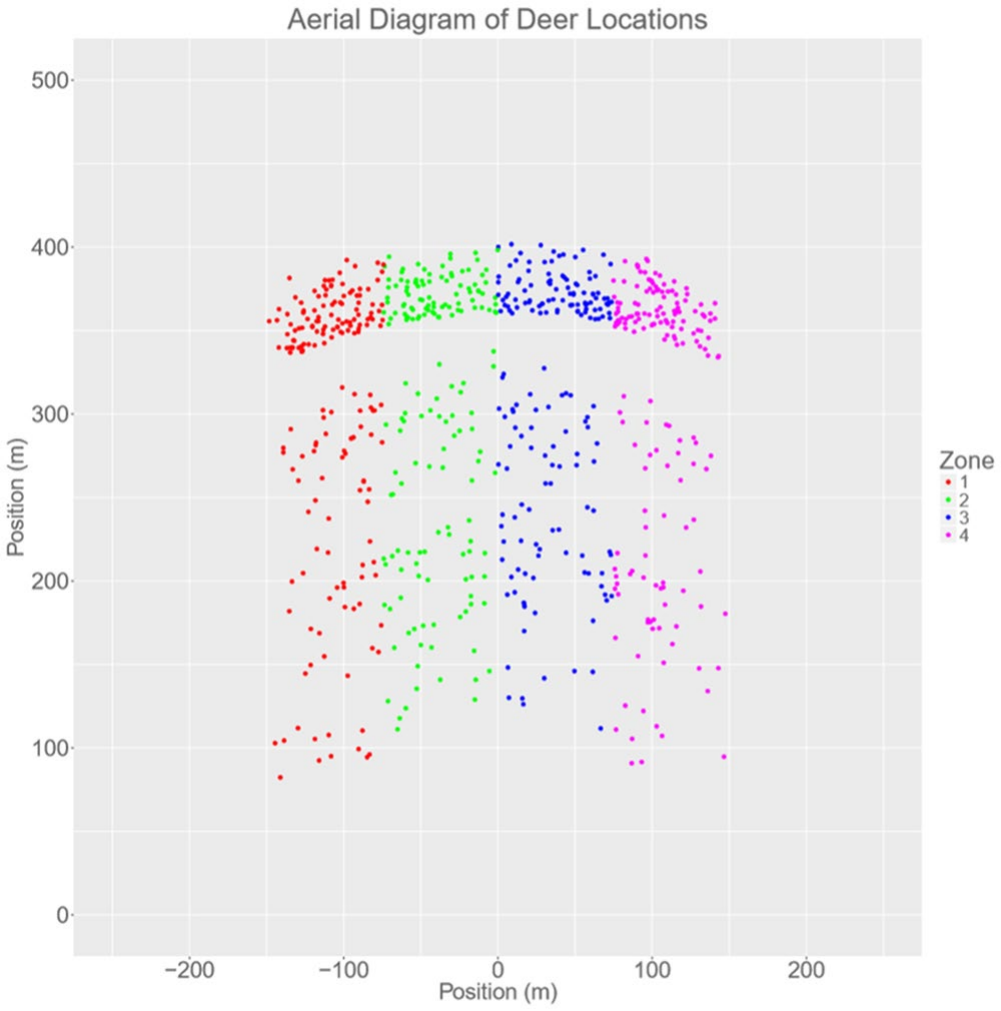
Aerial diagram of deer positions for all trials. The participant start position is (0, 0) then during the trial participants moved along the Y-axis. The 4 trials in each environment type and density combination each used a different zone to ensure that the deer would never be in roughly the same place each time. The clear thick band towards the upper part of the figure is all the max-distance and lowest density grassland deer start positions.

By combining the factors of environment type (grassland, wooded), density (2000, 5000, 8000, 11000 vegetation objects per km^2^) and deer start distance strategy (max distance, non-max distance) we arrived at 16 combinations in total. Each was repeated twice giving a total of 32 trials. This meant that participants have seen each environment type and vegetation combination 4 times. To ensure an even distribution of deer placement locations across the different conditions we used a system of zones when placing the deer (Fig. 5). Each trial lasted for two minutes on average, meaning the experiment (per participant) lasted for about one hour.

#### Participants

22 participants took part in the experiment (5 male, 17 female, mean age 20.6 years, SD = 3.414). All participants gave informed consent before taking part. Some participants were recruited via the Psychology Department’s participant pool and received course credits for their participation, whereas participants from other University Departments received no compensation. Each participant was provided with a written description of what would happen during the experiment and had the opportunity to ask questions.

The use of modern human individuals today in an experiment to infer behavioural traits in the deeper past needs addressing. In the case of the Late Pleistocene modern humans (*Homo sapiens*), the justification may be more easily made than for Neanderthals (*H*. *neanderthalensis*) because of relative taxonomic continuity and presumed similarities in cognitive and brain functional traits shared between modern humans today and in the Pleistocene. We assume that our participants are an appropriate proxy for the anatomically modern human (AMH) populations of MIS3 in terms of the visual processing system (and we are not aware of any differences in visual processing between populations alive today). It may be, however, that applying the results of the experiments conducted here to Neanderthals is less problematic than might appear. While it has been suggested that Neanderthal vision may have been different in for example colour perception, edge detection and visual acuity, and that they were better at seeing in low light levels^24^, there is still likely to have been a gradient of prey-detecting abilities according to vegetation structure, although it may have been different to that in AMH.

#### Procedures

The participants completed the 32 trials in a randomised order. They were required to click an on-screen prompt to begin a trial, which allowed them to take a short break whenever they desired.

When a trial started, the participant was passively transported in a straight line along the centre of the environment towards the opposite end of the environment, gradually reducing the distance between the participant’s viewpoint and the deer. The energetic cost of transport for human walking is minimised at speeds ranging from 5–6 km/hr^25^, so in our experiments the participant was moved forwards at 5.4 km/hr (or 1.5 m/s, which corresponds to brisk walking). The participant had been instructed to search for the deer and to press the space bar on the computer keyboard as soon as they found the deer. Movement then stopped, and participants were asked to indicate the position of the deer using the computer’s mouse. This final step ensured that the participant had really seen the deer when they claimed to.

If the participant’s viewpoint had moved so far forwards that it had passed the deer without the participant responding, the trial would be recorded as a non-response and a prompt appeared asking the participant to move on to the next trial. If the participant responded by indicating a location where there was no deer, this would be recorded as an incorrect response.

The participant was not told whether they correctly identified the deer location, they simply moved onto the next trial. If the participant indicated that they spotted the deer, the distance from the participant to the deer was recorded at the time of response whether the participant was correct in locating the deer or not.

The position of any 3D model within a virtual world is typically expressed as a single point location, around which the model will occupy some volume of 3D space. The point location of the model may also be expressed in screen coordinates, typically measured in pixels from the top left corner of the screen. The 3D model itself can then be thought of as “occupying” a certain number of pixels around this screen location. The deer model occupied fewer pixels the further away from the participant viewpoint it was, but occupied at least a 50 pixel radius around its screen location at even the furthest distances used in the experiment. We recorded how far away in pixels the participant had clicked from the screen coordinates of the deer, and responses that were within 50 pixels of the deer model’s screen coordinates were automatically classed as correct. Every response over 250 pixels away from the model’s screen coordinates was flagged as incorrect, as this is far enough away that the participant must not have clicked on the deer unless it was very close to their viewpoint, which none were. The correctness of responses between these thresholds was determined manually by later recreating the scene from the participants’ viewpoints at the time of response to see whether the mouse pointer was over the deer model when they responded.

### Experiment 2

The aim of the first experiment was to test two different distinct biomes (grassland and wooded) featuring different vegetation densities. However, real-life environments need not necessarily fit neatly into either biome. This is particularly true if we are considering an environment that is transitioning between the two biomes over time. For an environment experiencing climatic cooling, the transition would be towards a more open grassland environment, whereas a warming environment would become more wooded and closed. Since this kind of environmental transition represents the conditions that European Palaeolithic societies experienced, we built on the first experiment by investigating how prey detection rates would have been affected by such a transition.

#### Materials

Participants again interacted with an Unreal Engine 4-based desktop application which presented 3D virtual environments via a 3-screen setup in the Bournemouth University Psychology Department. The same 3D models of flora and deer were used as in Experiment 1.

#### Participants

20 participants took part (10 male, 10 female, mean age 21.15 years, SD = 3.70). All participants gave informed consent before taking part. Some participants were recruited via the Psychology Department’s participant pool and received course credits for their participation, whereas participants from other University Departments received no compensation. Each participant was provided with a written description of what would happen during the experiment and had the opportunity to ask questions.

One participant did not give a single correct response in any of the three most wooded environments and had only responded correctly in 5 of their 15 trials overall. This made it impossible to calculate a mean prey detection distance for them in those three environments and indicates that they may have had difficulty in completing the task, or misunderstood the task. Their data were excluded from further analysis.

#### Procedures

**Figure 6:**
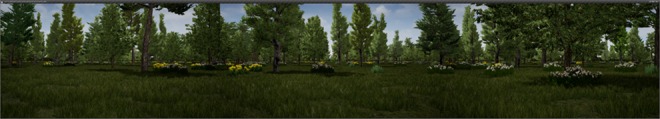
Typical virtual environment of mixed wooded and grassland flora as used in Experiment 2.

The environments presented varied along a spectrum from grassland to wooded. Environments always contained 11,000 vegetation objects per km^2^ but they were a mixture of wooded environment taxa (trees) and grassland environment taxa (shrubs, see Fig. 6). As in Experiment 1, each environment also contained 50 rocks (each ~180 cm tall) at a density of 200 per km^2^.

The deer was again present in all trials. There were 5 levels of environment composition (vegetation consisting of 10%, 30%, 50%, 70%, or 90% wooded taxa, with the remainder being grassland taxa). Three trials were conducted in each level, providing a total of 15 trials per participant. The deer was at least 360 m away from the participant start position, which was equal to the highest spotting distance obtained from any environment in Experiment 1 that was at the same density as the environments to be used in this experiment.

The participants were presented with the 15 trials in a completely random order. They were required to press a key to begin the next trial so they could take a short break whenever they required.

## Results

We used R^26^ for all statistical analysis (version 3.5.2) and the lme4^27^ package to carry out linear mixed effect modeling.

### Experiment 1

#### Correct responses

Any individual trial ended with one of three outcomes; first, the participant responded correctly (found the deer), second, the participant responded incorrectly (indicated a position where there was no deer), or third, the participant did not respond at all and the trial ended when they had passed the deer.

Overall, participants responded correctly in 90.20% of trials, incorrectly in 7.53%, and gave no response in 2.27%. Every participant gave at least one correct response in each environment type and density condition combination. A breakdown of response type by environment type is presented in the Supplementary Information (Table S1).

In the further analysis we focus on the correct responses. For each correct response we calculated the distance in meters between the participant in the virtual environment and the deer when the response was given. We refer to this measure as the “prey detection distance”.

#### Effects of environment type and vegetation density

We ran a linear mixed effect (LME) analysis for prey detection distance. Fixed effects were the environment type as a factor (wooded, grassland), and vegetation density as a continuous variable (2000 to 11000, rescaled to be in the range −1 to +1). Random factors were participant, and deer placement strategy (max distance, non-max distance). Akaike Information Criterion (AIC) was used to gauge the fit of the model. We started with an intercept only model and added by-participant slopes for the fixed effects and the interaction between them one by one. We found that none of these additional models significantly improved upon the intercept only model presented in Table 1.

**Table 1:** Results of LME analysis on data obtained during Experiment 1 from trials in which the participant successfully located the deer within the environment.

*Predictor of Prey Detection Distance* (*m*)	*Coefficient*	*SE*	*t statistic*
(Intercept)	159.87	8.92	17.92
Environment Type: Grassland	53.98	3.93	13.72
Density	−40.41	3.75	−10.77
Environment Type: Grassland × Density	−14.67	5.31	−2.77

We found reliable effects for both environment type and vegetation density as well as a reliable interaction between them. The effect of environment type was more pronounced when vegetation density was lower.

To investigate whether our deer placement strategy was affecting the results we ran a similar analysis without the random effect of placement strategy on only the trials that utilised the maximum distance placement strategy, and then again on the other half of the data. These analyses each yielded very similar trends as the data given in Table 1 (see Supplementary Information, Tables S2, S3), indicating that our approach was robust.

#### Mean prey detection distance

**Figure 7:**
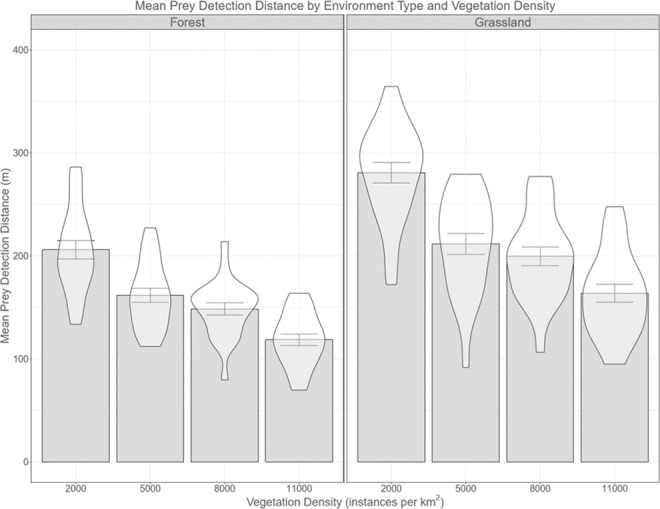
Bar and violin plots of mean prey detection distance by environment type and density. The mean prey detection distance decreases with increasing vegetation density and is lower for a wooded environment when compared with a grassland of the same density. Error bars are standard error of mean.

In agreement with our hypothesis, we found that the mean prey detection distance decreased as the vegetative density increased and it was always lower in wooded environments than in grassland environments of the same density (Fig. 7). Specifically, the mean prey detection distance was greater overall in the grassland environments (214.66 m) than in the wooded environments (160.29 m), and decreased from 242.92 m in the least dense environments to 140.72 m in the most dense.

### Experiment 2

#### Mean prey detection distance

**Figure 8:**
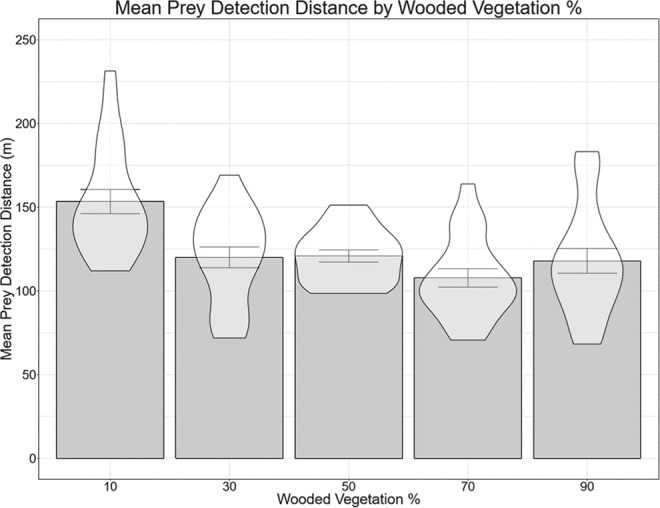
Mean prey detection distance by wooded vegetation percentage. Error bars are standard error of mean. Mean prey detection distance fell from ~150 m in the most open environment to ~107–120 m in all others. In particular, the 30%, 50%, 70% and 90% wooded environments all gave very similar mean prey detection distances.

Participants responded correctly in 88.25% of trials, a breakdown of response type by Environment Wooded Percentage is given in the Supplementary Information (Table S4). As with Experiment 1, the mean prey detection distance was plotted for each environment type (Fig. 8). We found that that the mean prey detection distance remained similar for all environments featuring wooded vegetation percentage of 30% or higher (around 120 m), although the 70% wooded environment features a lower mean prey detection distance than the others (107.80 m). The least wooded environment (wooded vegetation 10%) however had a mean prey detection distance of 153.38 m, around 30 m higher than any of the other environments.

To investigate this apparent non-linear relationship between mean prey detection distance and wooded vegetation percentage in more detail, we ran two LMEs for prey detection distance with wooded vegetation percentage as a fixed effect and the participant as a random effect. The first model included all levels of wooded vegetation percentage and revealed a reliable effect of wooded vegetation percentage on prey detection distance (see Table 2A). The second model was run on a subset of the correct response data which excluded the 10% wooded vegetation environments and did not reveal a significant effect (see Table 2B). These results support the notions that; (1) prey detection distance did not change between 30–90% wooded vegetation and (2) that there was a non-linear relationship between wooded vegetation percentage and mean prey detection distance.

**Table 2:** Results of the series of LMEs performed to investigate the effect of wooded vegetation percentage on mean prey detection distance.

Predictor of prey detection distance (m)	*Coefficient*	*SE*	*t statistic*	*AIC*
A (All correct trials)				2839.6
(Intercept)	145.08	5.55	26.15	
Wooded Vegetation Percentage	−0.41	0.10	**−4**.**22**	
B (wooded vegetation percentage >10)				2197.6
(Intercept)	124.17	8.21	15.12	
Wooded Vegetation Percentage	−0.11	0.13	−0.89	
C (log transformed prey detection distance)				293.2
(Intercept)	4.92	0.05	96.7	
Wooded Vegetation Percentage	−0.004	0.0009	**−4**.**10**	
D (square root transformed prey detection distance)				1164.7
(Intercept)	11.88	0.25	46.86	
Wooded Vegetation Percentage	−0.02	0.004	**−4**.**27**	

The notion of a non-linear relationship is further supported by a series of t-tests which showed that mean prey detection distance was significantly different in the 10% wooded environments from all other environments, while performance in the 30–90% wooded environments did not differ from each other. These analyses are presented in the Supplementary Information (Table S5).

To further investigate the nature of the non-linear relationship between prey detection distance and percentage wooded vegetation we ran two further LMEs in which we either log transformed or square root transformed prey detection distance (Table 2C,D). Comparing all four LMEs, the one in which the prey detection distance was log transformed resulted in the lowest AIC value. In other words, of the relationships assessed here, our data fits best with an exponential decline of prey detection distance with increasing percentage of wooded vegetation.

## Discussion

In this study we presented two experiments that utilised virtual worlds to investigate the influence of biome type and vegetative density on the distance at which participants could accurately locate a prey animal. The prey animal in this case was a female red deer, and the biome types used were grasslands and wooded areas.

The data obtained in Experiment 1 indicate that the distance at which prey can be reliably located decreases as the density of vegetation in an environment increases, and that given the same vegetation density, mean prey detection distances will be reliably higher in a more open grassland environment as compared to a more closed wooded environment. The data also shows that there is not a sharp cut-off point where prey detection performance changes drastically. This suggests that, as a real environment undergoes gradual change in density of vegetation, a population of hunters living there may not experience sudden changes in their circumstances as far as prey detection is concerned.

The decrease in participants’ performance as vegetation density increases was not unexpected as this is likely to be a combination of the well-studied *distractor effect*^18^, where increasing the number of objects means there are more things for the target to be lost amongst although technically fully visible, and also that increasing the number of vegetation objects increases the likelihood of the target either being occluded or completely hidden from view.

Experiment 1 also indicates that there are differences in prey detection performance when comparing an open area, such as grassland to a more closed wooded area of the same vegetation density. This could be caused by the fact that trees can completely hide the deer from view, whereas bushes/shrubs cannot. Therefore, Experiment 2 was designed to investigate this effect further by utilising virtual environments composed of a mixture of the two biomes.

Experiment 2 suggests that as an environment transitions from grassland to wooded at the same density (defined by differing relative proportions of shrub vs. tree vegetation), there is a non-linear change in mean prey detection distance. In our virtual environments, changes in prey detection distance may be explained by an exponential decay function with rapid decline between 10% and 30% wooded vegetation and much weaker decline with increasing percentages of wooded vegetation. In the real-world, the change in prey detection ability may occur once a different relative mix of vegetation taxa is reached, depending on biome. If this effect is genuine, it may help explain the environmental pressures of such transitions on hominin populations as climate and accompanying vegetation changes. For example, a hunting strategy which relies on being able to locate prey at greater distances, like pursuit hunting, may become viable relatively quickly once an environment reaches a certain composition. Alternatively, hunting gradually becomes more viable during the course of the entire transition from wooded to grassland.

Although no contemporary environment analogous to the open steppe-tundra of cooler MIS3 Europe contains hunter-gatherers, very open environments with different climatic conditions exist today where hunting is still practiced. Persistence hunting (or pursuit hunting), in which animals are run to exhaustion before being killed by hunters has been argued to have been favoured in open environments^12^. Rather than persistence hunting, the procurement strategies employed by Palaeolithic humans in closed wooded biomes is thought to have included encounter hunting strategies^28^ or the preparation of specialised weapons, hunting groups, and careful timing of the movement of human groups^29^. As persistence hunting clearly relies on the ability to locate prey animals at a distance, we argue that this higher degree of prey visibility plays a major role in determining whether persistence hunting is a viable strategy to use.

We know that vegetation varies between open and closed as climatic shifts occur, but it is unclear what effect this would have on hunting styles, as dissimilar hunting strategies are usually thought of as being discreetly different. If a hominin population is to remain in the same location, then a significant change in hunting behaviour may be required if the hunting strategy utilised before the environment changed is not as effective afterwards due to changes in prey detection performance.

During the Quaternary, and MIS3 in particular, many hominin population turnovers are known to have occurred^7,30^. A significant turnover during MIS3 is when the Neanderthals were replaced by anatomically modern humans (AMH). This transition has been variously explained as due to climate change and the associated ecological change and/or competition from the newly-arrived AMH^6,31^. Ancient DNA analysis of AMH remains has revealed the relatedness of their populations across the broad region of Eurasia during MIS3^32^. This has shown that all Europeans living between ~37,000 and ~14,000 years before the present (BP) came from a single founding population (associated with the Aurignacian culture) that arrived at around 37,000 years BP and persisted through the Late Pleistocene^32^. This population had variation in different parts of Europe, one of which seems to have been displaced around 33,000 years ago (Věstonice Cluster) associated with the transition to the Gravettian. At around 19,000 years BP, a population more related to the first population re-expanded across Europe, possibly from the south-west and associated with the Magdalenian^32^. The final event was the “major population turnover” at around 14,500 years BP^33^. This turnover, involving a new population coming from the east, was perhaps associated with the Epigravettian^32,33^, and possibly occurred during a major biome change caused by the Bolling-Allerod interstadial complex^34^. Evidence obtained from ice cores suggests rapid warming occurred prior to the onset of this period, with average July temperatures rising by 4–5 °C within half a century^35,36^, leading to changes in the distribution of vegetation across Europe^37^. Human populations living in Europe during parts of MIS3 could have experienced temperature fluctuations of 5–8 °C over the course of 500–2000 years due to Dansgaard-Oeschger events^23^, and would have also experienced changes in the distribution of vegetation that would have accompanied such a shift in climate^5^. However, the changes may have been faster when moving from interstadials to stadials than the other way around due to the limitations of tree migration rates.

These populations living through the climatic upheavals of MIS3, the LGM or the Bolling-Allerod may not have experienced pressure to modify their hunting behaviours until the changing vegetative environments in which they lived reached specific compositions and densities, leading to changes in prey detection ability. For example, during the Bolling-Allerod the vegetation became more wooded as global climates warmed. This warming is well known to have caused a change in mammalian ecology leading to the loss of megafauna in Europe^38^. Another effect would have been the increase in distractor numbers (trees) causing a change in prey detection by modern humans. Following such prey detection changes there could have been pressure to either adapt their behaviours to suit their new circumstances, or to move on to areas more suitable to their existing behaviours, or, finally, to face local extinction. This could be of principal importance for hominin populations whose hunting techniques are thought to favour specific biomes e.g. the close-range hunting style thought to have been used by Neanderthals^39^.

It should also be considered, however, that just as there is no clean and strict division between grassland and wooded environments, it should not be expected that there is a division between situations where one hunting technique would have been exclusively suitable. While we know that vegetation will vary on a gradient, whether linearly or in a punctuated fashion^4,40^, it is unclear how hominin hunting styles would need to vary across the spectrum of vegetation. It is likely, however, that the effects of vegetation structure on prey visibility will have a knock-on effect on hunting styles.

These experiments suggest that an environmental transition in which the relative mix of grassland / wooded vegetation remains similar, but vegetative density is changed, may have a more gradual effect on prey detection ability than a transition during which the environment becomes either more open or closed. Specifically, during the latter type of transition, prey detection ability may be mostly unaltered until a crucial point in the transition, after which hunting success may be affected over a shorter period of time. The latter may subsequently have had an important selective effect on a population’s viability in a specific environment.

### Ethics

The experiments detailed in this article were approved by the Bournemouth University Ethics Programme Team. All participants gave informed consent before taking part, and could withdraw at any time. All experiments were performed in accordance with the relevant guidelines and regulations.

## Supplementary information

Supplementary Information

## Data Availability

The raw data which was collected and analysed in Experiment 1: https://www.dropbox.com/s/em7pf8qv9ro4ilx/Experiment%201%20Data.zip?dl=0. The raw data which was collected and analysed in Experiment 2: https://www.dropbox.com/s/hs1ovzcwm1l0ru0/Experiment%202%20Data.zip?dl=0.
