# Supplementary material for: Investigating the Effect of the Environment on Prey Detection Ability in Humans
Source: Sci Rep. 2019 May 15;9:7445. doi: 10.1038/s41598-019-43797-0 (PMC6520383; doi:10.1038/s41598-019-43797-0)
Supplement: Supplementary file 1 — Supplementary Information [file 41598_2019_43797_MOESM1_ESM.docx]

# Investigating the Effect of the Environment on Prey Detection Ability in Humans.

# Supplementary Information

Peter J Allen*^1^, Jan M Wiener^2^, Christos Gatzidis^1^, Chris B Stringer^3^, John R Stewart^4^

^1^ Department of Creative Technology, Bournemouth University, Talbot Campus, Fern Barrow Poole, Dorset BH12 5BB, UK.

^2^ Department of Psychology, Ageing and Dementia Research Centre, Bournemouth University, Talbot Campus, Fern Barrow Poole, Dorset BH12 5BB, UK.

^3^ Department of Earth Sciences, Natural History Museum, London SW7 5BD, UK.

^4^ Department of Life and Environmental Science, Faculty of Science and Technology, Bournemouth University, Talbot Campus, Fern Barrow Poole, Dorset BH12 5BB, UK.

Corresponding author: Peter Allen: [allenp@bournemouth.ac.uk](mailto:allenp@bournemouth.ac.uk)

# Supplementary Information

Table S1 - Experiment 1 response type breakdown

| Density  (veg. objects per km^2^) | Environment | Correct Response (%) | Incorrect Response (%) | Non-Response (%) |
| --- | --- | --- | --- | --- |
| 2000 | Wooded | 88.64 | 11.36 | 0 |
|  | Grassland | 97.72 | 2.27 | 0 |
|  | All | 93.18 | 6.81 | 0 |
| 5000 | Wooded | 84.10 | 13.64 | 2.27 |
|  | Grassland | 90.91 | 9.09 | 0 |
|  | All | 87.50 | 11.36 | 1.14 |
| 8000 | Wooded | 81.82 | 13.64 | 4.55 |
|  | Grassland | 88.64 | 6.82 | 4.55 |
|  | All | 85.22 | 10.23 | 4.55 |
| 11000 | Wooded | 84.09 | 9.09 | 6.82 |
|  | Grassland | 84.09 | 13.64 | 2.27 |
|  | All | 84.09 | 11.36 | 4.55 |

Table S2 - The results of the Experiment 1 LME analysis when performed only on the half of the correct response data from which utilised the max distance deer placement strategy are presented below. The overall trends displayed in this data is the same as when the whole dataset is analysed.

| *Predictor of Mean Prey Detection Distance (m)* | *Coefficient* | *SE* | *t statistic* |
| --- | --- | --- | --- |
| (Intercept) | 165.56 | 7.52 | 22.00 |
| Environment Type: Grassland | 58.70 | 5.89 | 9.98 |
| Density | -32.48 | 5.65 | -5.75 |
| Environment Type: Grassland x Density | -18.75 | 7.88 | -2.38 |

Table S3 - Similar analysis as presented in Table S2, but performed on the other half of the Experiment 1 data (non-max distance trials), again displaying the same trends as the overall dataset.

| *Predictor of Mean Prey Detection Distance (m)* | *Coefficient* | *SE* | *t statistic* |
| --- | --- | --- | --- |
| (Intercept) | 154.09 | 6.27 | 24.57 |
| Environment Type: Grassland | 49.73 | 5.24 | 9.50 |
| Density | -47.93 | 4.98 | -9.63 |
| Environment Type: Grassland x Density | -11.14 | 7.13 | -1.56 |

Table S4 - Experiment 2 Response type breakdown.

| Environment Wooded % | Correct % | Incorrect % | Non-response % |
| --- | --- | --- | --- |
| 10 | 95.24 | 3.17 | 1.59 |
| 30 | 93.65 | 0 | 6.34 |
| 50 | 85.71 | 3.17 | 11.11 |
| 70 | 80.95 | 3.17 | 15.87 |
| 90 | 85.71 | 4.76 | 9.52 |

Table S5 - Experiment 2 Pairwise t-tests. Comparisons of mean prey detection distances between the least wooded and all other environments. There were no statistically significant comparisons between the four 30%+ wooded environments and each other.

| Environment Closed (%) | 10 |
| --- | --- |
| 30 | t(106) = -3.22, p < 0.01, d = 0.60 |
| 50 | t(99) = -3.83, p < 0.01, d = 0.72 |
| 70 | t(103) = -4.90, p < 0.001, d = 0.92 |
| 90 | t(109) = -3.29, p < 0.001, d = 0.62 |
